# Recent Trends in the Prevention and Management of Obesity Among Adults: A Systematic Review

**DOI:** 10.7759/cureus.89207

**Published:** 2025-08-01

**Authors:** Gauri Shankar, Jitender Sharma, Rahul Soni, Shrey Gondalia, Vinod Kumar

**Affiliations:** 1 Medical Writing, Index Research International, Vadodara, IND; 2 Neurology, Base Hospital Delhi Cantt, New Delhi, IND; 3 Medicine, Jagruti Hospital, Jamnagar, IND; 4 Surgery, Shaikh-Ul-Hind Maulana Mahmood Hasan (SMMH) Government Medical College, Saharanpur, IND

**Keywords:** adult obesity, obesity, obesity management, overweight, weight loss

## Abstract

Obesity has become a challenging public health concern because of its several health consequences. This systematic review aimed to explore recent trends in the prevention and management of obesity among adults. This systematic review was conducted following the guidelines of the Preferred Reporting Items for Systematic Reviews and Meta-Analysis (PRISMA). The literature search encompassed extensive databases like PubMed, ScienceDirect, and Cochrane Library for the last ten years, from 2015 to 2025. The quality of the included studies was evaluated using the appropriate tools according to the study design. The synthesis and data analysis included a summary of study characteristics, interventions, outcomes measured, and main study results/conclusions. Sample sizes in the included studies ranged from 21 to 4047 participants. Pharmacological interventions, such as glucagon-like peptide-1 receptor agonists, demonstrated significant and sustained weight loss and improved cardiometabolic risk factors. Exercise interventions were shown to improve aerobic capacity, body composition, and blood pressure. Time-restricted eating, especially when combined with resistance training, proved effective for fat loss and maintaining muscle mass. Bariatric surgery resulted in significant weight loss, increased diabetes remission, and reduced incidence of diabetes-related complications. Additionally, the use of probiotics may aid in weight management by controlling body fat mass and reducing waist circumference. Combination therapies, such as weight loss programs incorporating both aerobic and resistance exercise, were found to be the most effective in reducing ectopic fat and improving physical and metabolic function. This systematic review highlights the multifaceted nature of obesity prevention and management among adults, emphasizing the effectiveness of diverse interventions like pharmacological agents, exercise therapy, dietary management, and surgical management such as bariatric surgery.

## Introduction and background

Obesity has emerged as one of the most significant public health challenges globally, with its prevalence rising at an alarming rate over the past few decades. According to the World Health Organization (WHO), worldwide adult obesity has more than doubled since 1990, and as of 2022, approximately 1 in 8 people globally were living with obesity [1]. For adults, WHO defines overweight as a BMI greater than or equal to 25 kg/m² and obesity as a BMI greater than or equal to 30 kg/m² [1]. This rapid increase has profound implications for health systems, economies, and societies at large, necessitating urgent attention to prevention and management strategies.

The global burden of obesity continues to escalate, with projections indicating that by 2025, the prevalence of obesity will reach 18% among men and exceed 21% among women worldwide [2]. In the United States, recent data from the National Health and Nutrition Examination Survey revealed that the prevalence of obesity among adults was 40.3% during 2021-2023, with severe obesity affecting 9.4% of adults [3]. Notably, obesity prevalence is highest among adults aged 40-59 years (46.4%) compared to younger and older age groups, and severe obesity is more prevalent in women than men across all age categories [3]. Similar to the United States, the overall prevalence of obesity among Indian adults is 40.3%, according to a study conducted by Venkatrao et al. (2020) [4]. According to the National Family Health Survey-5 (2019-2021) data published in 2023, 23% of women and 22.1% of men are overweight in India [5].

These epidemiological trends are not confined to high-income countries; middle- and low-income countries are experiencing rapid increases in obesity prevalence, often alongside persistent undernutrition, creating a double burden of malnutrition [6]. The World Obesity Atlas 2023 projects that by 2035, over half of the global population will be overweight or obese, with certain regions such as the South Pacific Islands facing particularly severe burdens [6].

Obesity is a complex, multifactorial chronic disease characterized by excessive accumulation of adipose tissue resulting from an imbalance between energy intake and expenditure. Obesity results when calorie intake exceeds calorie expenditure for any reason, such as a sedentary lifestyle, reduced physical activity, or excessive consumption of a high-calorie diet. Beyond simple caloric imbalance, obesity involves intricate interactions among genetic, metabolic, environmental, behavioral, and psychosocial factors [1,6]. Adipose tissue in obesity undergoes pathological remodeling, including hypertrophy and hyperplasia of adipocytes, leading to altered secretion of adipokines and pro-inflammatory cytokines. This chronic low-grade inflammation contributes to insulin resistance, endothelial dysfunction, and metabolic dysregulation [1].

Central to the pathophysiology is the role of visceral adiposity, which is metabolically active and strongly linked to adverse cardiometabolic outcomes. Excess visceral fat promotes lipotoxicity, oxidative stress, and systemic inflammation that impair insulin signaling pathways, thereby increasing the risk of type 2 diabetes mellitus (T2DM) and cardiovascular disease (CVD) [3]. Additionally, obesity disrupts neuroendocrine regulation of appetite and energy balance, involving hormones such as leptin, ghrelin, and insulin, which further complicates weight management efforts [1].

There are several detrimental health consequences of obesity, which are well-documented. Obesity significantly increases the risk of multiple chronic diseases, including hypertension, T2DM, coronary heart disease, stroke, certain cancers (such as breast, colorectal, and endometrial cancers), osteoarthritis, and respiratory conditions like obstructive sleep apnea [1,3]. The presence of obesity also exacerbates the severity and progression of these diseases, resulting in increased morbidity and mortality.

Metabolic syndrome, characterized by a cluster of risk factors including abdominal obesity, dyslipidemia, hypertension, and hyperglycemia, is highly prevalent among individuals with obesity and substantially elevates cardiovascular risk [1]. Moreover, obesity is associated with impaired quality of life, mental health disorders such as depression and anxiety, and increased healthcare utilization and costs [6]. In 2021, an estimated 3.7 million deaths from noncommunicable diseases were attributed to a higher-than-optimal BMI. These diseases include CVD, diabetes, cancers, neurological disorders, chronic respiratory diseases, and digestive disorders [7].

Despite the substantial burden of obesity and its complications, effective prevention and management remain challenging. The multifactorial etiology of obesity necessitates comprehensive approaches that integrate lifestyle modification, pharmacotherapy, and surgical interventions tailored to individual needs. Recent advances in pharmacological treatments, novel dietary patterns such as time-restricted eating (TRE), and optimized exercise regimens have shown promise in improving outcomes. Additionally, emerging evidence suggests the potential role of probiotics and combination therapies in enhancing weight loss and metabolic health.

However, there is a need to systematically synthesize and critically evaluate these recent trends to inform clinical practice and public health policies. Understanding the comparative effectiveness, safety profiles, and long-term impacts of various interventions is essential to guide personalized treatment strategies and optimize resource allocation.

This systematic review aims to explore the latest evidence on prevention and management strategies for adult obesity, focusing on pharmacological, surgical, dietary, exercise, and adjunctive therapies. By consolidating current knowledge, this study seeks to identify gaps, inform future research directions, and support evidence-based decision-making in combating the obesity epidemic.

## Review

Methodology

This systematic review adhered to the 2020 guidelines for Preferred Reporting Items for Systematic Reviews and Meta-Analyses (PRISMA) [8].

Search Sources and Strategy

Comprehensive literature searches were performed across several electronic databases, including PubMed, ScienceDirect, and Cochrane Library, for the last ten years, from 2015 to 2025. The search strategy incorporated relevant keywords like “adult obesity”, “overweight”, “pharmacological management”, “surgical management”, “exercise therapy”, “diet”, and “lifestyle modification” along with corresponding Medical Subject Headings (MeSH) terms such as “Obesity”, “Overweight”, “Pharmacologic Therapy”, “Bariatric Surgery”, “Exercise Therapy”, “Diet Therapy”, and “Lifestyle”. Boolean operators ‘AND’ and ‘OR’ were applied to combine these terms effectively, for example: (“Obesity” OR “Overweight” OR “adult obesity”) AND (“Pharmacologic Therapy” OR “Bariatric Surgery” OR “Exercise Therapy” OR “Diet Therapy” OR “Lifestyle” OR “pharmacological management” OR “surgical management” OR “exercise therapy” OR “diet” OR “lifestyle modification”).

Eligibility Criteria

Eligibility criteria included studies involving adults aged 18 years and above with overweight or obesity, focusing on interventions such as pharmacological management, surgical management, exercise therapy, diet, and lifestyle modification. Only studies published in English within the last ten years (2015-2025) were considered, encompassing randomized controlled trials (RCTs), prospective, and observational studies. We excluded systematic reviews, meta-analyses, case reports, case series, short communications, commentaries, and editorials. Animal studies were also excluded.

Study Selection

After removing duplicates, two independent reviewers screened titles and abstracts to identify potentially eligible studies. Non-English articles, animal studies, and those not meeting the inclusion criteria were excluded. Full texts of eligible articles were retrieved and assessed independently by the reviewers. Inconsistencies were resolved through either collaborative discussion or by seeking input from a third, independent reviewer.

Data Extraction and Data Synthesis

After removal of duplicates, two independent reviewers screened titles and abstracts for relevance, followed by full-text assessments to confirm eligibility. Data extraction and synthesis were conducted using a standardized form capturing study characteristics, designs, sample sizes, intervention details, outcomes, and quality assessments. This methodology ensured a rigorous and systematic approach to reviewing the literature on adult obesity prevention and management, incorporating key search terms, Boolean operators, and adherence to PRISMA guidelines for high-quality evidence synthesis.

Risk of Bias Assessment

We critically appraised the methodological quality of included studies using the Cochrane Risk of Bias tool for RCTs, which evaluates seven domains: random sequence generation, allocation concealment, blinding of participants and personnel, blinding of outcome assessment, incomplete outcome data, selective reporting, and other biases [9]. Studies were categorized as having a low, high, or unclear risk of bias based on these criteria. The prospective study was evaluated using the Risk of Bias in Non-randomized Studies of Interventions (ROBINS-I) tool [10]. Two independent reviewers conducted the assessments, and disagreements were resolved by consensus.

Results

**Figure 1:**
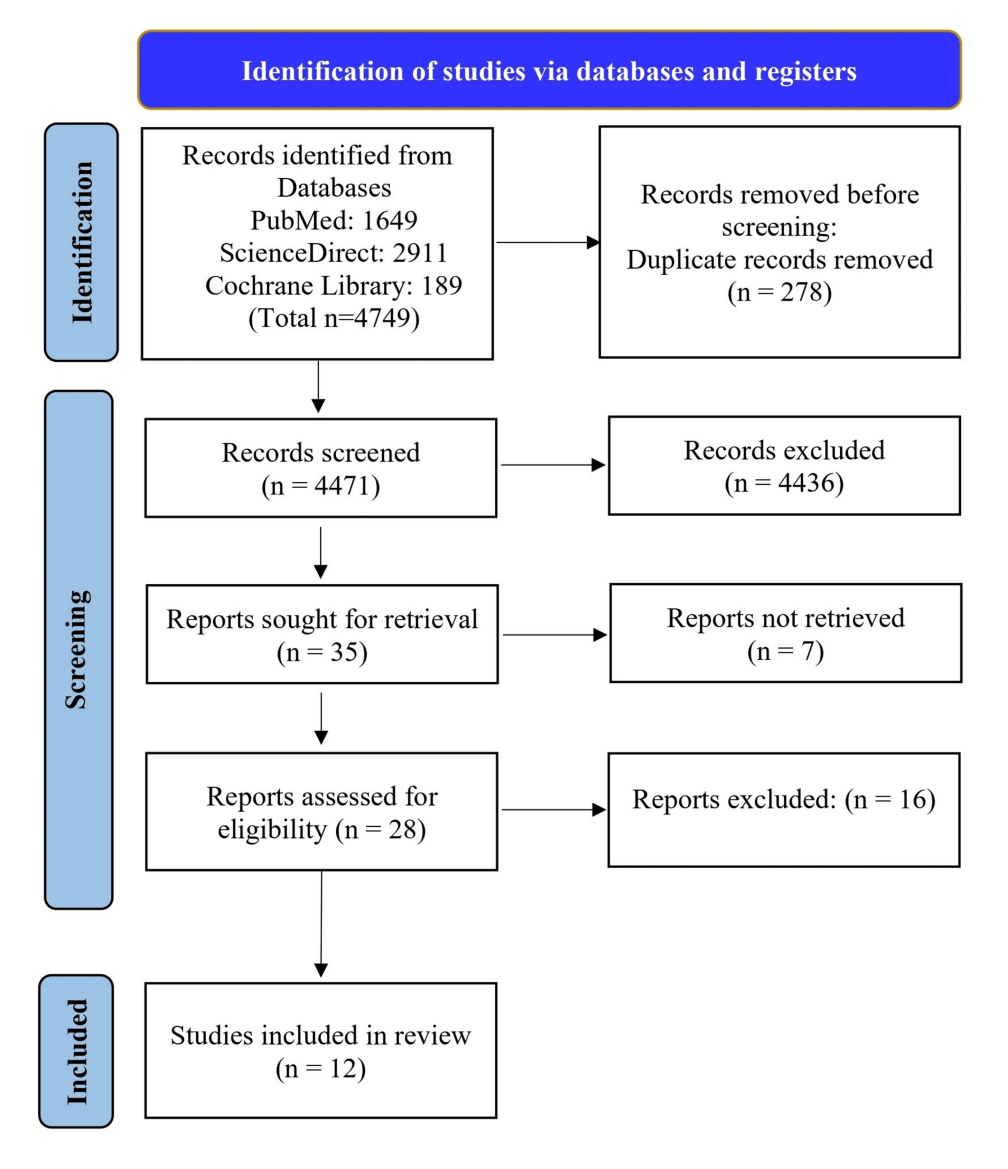
PRISMA flow diagram PRISMA: Preferred Reporting Items for Systematic Reviews and Meta-Analyses

The initial search yielded 4749 studies from different databases; 4471 records were screened after the initial exclusion of the studies and removal of duplicates. Following an assessment of the titles and abstracts, 35 articles were selected for further consideration. Following that, seven studies were eliminated based on the inclusion criteria. We screened 28 studies based on the prescribed inclusion and exclusion criteria. Finally, we synthesized 12 studies for qualitative analysis because of the unavailability of some data in the other studies. The process of selection of the studies is illustrated in the PRISMA study selection diagram (Figure 1).

Study Characteristics

Table 1 summarizes a diverse range of interventional studies [11-22] investigating various lifestyles, pharmacological, surgical, and dietary strategies for weight management and metabolic health. These studies span multiple countries and include predominantly RCTs, with sample sizes ranging from 21 to 4047. The interventions examined include pharmacological agents such as liraglutide and semaglutide, TRE, probiotics, structured exercise programs, bariatric surgeries (e.g., Roux-en-Y gastric bypass (GB), endoscopic sleeve gastroplasty), and comprehensive lifestyle modifications.

**Table 1:** Characteristics of the included studies RCT: randomized controlled trial, TRE: time-restricted eating, RT: resistance training, MICT: moderate-intensity continuous training, HIIT: high-intensity interval training, LS: lifestyle, LU: polydextrose fiber, BMI: body mass index, DEXA: dual-energy X-ray absorptiometry, VO₂max: maximum oxygen uptake, SBP: systolic blood pressure, BP: diastolic blood pressure, MAP: mean arterial pressure, RMR: resting metabolic rate, EEDE: energy expenditure during exercise, VO₂peak: peak oxygen uptake, T1/2: half-time of gastric emptying, %TBWL: percent total body weight loss, hsCRP: high-sensitivity C-reactive protein, GLP-1: glucagon-like peptide-1, PYY: peptide YY, CFU: colony forming units, MRI: magnetic resonance imaging, CPET: cardiopulmonary exercise testing, OGTT: oral glucose tolerance test, PHQ-9: patient health questionnaire-9, SF-36: 36-item short form survey, PFS: physical functioning scale, TFEQ-18: 18-item three-factor eating questionnaire, CoEQ: control of eating questionnaire, ISI: insulin sensitivity index, IMAT: intermuscular adipose tissue, VAT: visceral adipose tissue, RYGB: Roux-en-Y gastric bypass, PELI: physical exercise and lifestyle intervention, ESG: endoscopic sleeve gastroplasty, T2DM: type 2 diabetes mellitus

Authors/year	Country	Study design	Sample size	Interventions used	Outcomes assessed	Conclusion of study
Pi-Sunyer et al., 2015 [11]	Multinational (27 countries of Europe, North America, South America, Asia, Africa, and Australia)	RCT	3731	Liraglutide 3.0 mg once daily subcutaneous injection plus LS counseling vs. placebo plus LS counseling	Change in body weight, proportion losing ≥5% and >10% body weight, BMI, waist circumference, glycemic control, cardiometabolic biomarkers, quality of life	Liraglutide 3.0 mg with diet and exercise significantly reduced body weight (mean loss 8.4 kg vs. 2.8 kg placebo), improved metabolic control, and was generally well tolerated over 56 weeks
Stenman, et al., 2016 [12]	Finland	RCT	225	(1) Placebo (microcrystalline cellulose, 12g/day). (2) LU (12g/day). (3) *Bifidobacterium animalis* ssp. lactis 420 (B420, 1×10¹⁰ CFU/day in 12g cellulose). (4) LU + B420 (12g + 1×10¹⁰ CFU/day)	Primary: relative change in body fat mass (by DEXA). Secondary: body weight, waist/hip circumference, food intake, blood and fecal biomarkers (e.g., serum zonulin, hsCRP), adverse events	B420 (with or without fiber) controlled body fat mass and reduced waist circumference and energy intake in overweight/obese adults (in per-protocol analysis). LU alone had no effect. No significant adverse events. Suggests probiotics may aid weight management
Chin, et al., 2020 [13]	Hong Kong	RCT	56	(1) Control (no intervention, n=14). (2) MICT 3x/week (n=9). (3) HIIT 3x/week (n=14). (4) HIIT 2x/week (n=10). (5) HIIT 1x/week (n=9) - MICT: 30 min at 60% HRR - HIIT: 12x1-min at 90% HRR + 11x1-min at 70% HRR per session	Aerobic capacity (VO2max, shuttle run distance). Body composition (body fat mass, fat-free mass, BMI, waist circumference). Blood pressure (SBP, DBP, MAP). Resting heart rate. Vascular function - fasting glucose, insulin, lipid markers	Even low-frequency (once weekly) HIIT improved cardiorespiratory fitness, body composition, and blood pressure in overweight/obese young men. Low-frequency HIIT is a feasible and effective initial exercise strategy for this population
Kristensson et al., 2020 [14]	Sweden	Prospective nonrandomized intervention study	4,047	Bariatric surgery (gastric bypass, banding, vertical-banded gastroplasty) vs. usual care	Body weight changes, T2DM remission and incidence, cardiovascular and microvascular disease incidence, energy intake, physical activity, mortality	Bariatric surgery benefits in adults are similar regardless of obesity status at 20 years of age; surgery leads to significant weight loss, increased diabetes remission, and reduced incidence of diabetes and microvascular complications independent of early or adult-onset obesity status. Cardiovascular outcomes and surgical complications were similar across subgroups
Berge et al., 2021 [15]	Norway	RCT	71	Two 24-week aerobic exercise programs: MICT vs. combined HIIT and MICT	Primary: EEDE. Secondary: RMR, cardiorespiratory fitness (VO2max), body composition (weight, BMI, fat mass, fat-free mass), waist circumference, appetite control	Both HIIT/MICT and MICT increased energy expenditure during exercise and induced weight loss; HIIT/MICT group lost on average 3 kg more weight but did not significantly increase EEDE compared to MICT alone. Tailoring exercise intensity to patients may be appropriate due to dropout rates in HIIT/MICT group
Kotarsky et al., 2021 [16]	USA	RCT	21	TRE with an 8-hour eating window (12 pm - 8 pm) vs. NE; both groups performed 8 weeks of aerobic and supervised RT	Primary outcomes: fat mass and fat‐free mass. Secondary outcomes: levels of physical activity, muscle performance, blood pressure, blood and saliva markers, and dietary intake	TRE combined with concurrent exercise training significantly reduced fat mass and increased lean mass in overweight and obese adults compared to normal eating. TRE is an effective short-term dietary strategy for fat loss and lean mass gain
Garvey et al. 2022 [17]	Multinational (Canada, Italy, Hungary, Spain, and the United States)	RCT	304	Once-weekly subcutaneous semaglutide 2.4 mg + behavioral intervention vs. placebo + behavioral intervention	% change in body weight at 104 weeks; proportion achieving ≥5% weight loss; waist circumference; blood pressure; glycemic status; lipids and safety/adverse events	Semaglutide 2.4 mg led to substantial, sustained weight loss (mean -15.2% vs -2.6% placebo at 104 weeks), improved cardiometabolic risk factors, and was well-tolerated. More GI side effects are seen with semaglutide. Supports long-term use in adults with overweight/obesity without diabetes
Waters et al., 2022 [18]	USA	RCT	160	(1) Control (no weight loss or exercise). (2) Aerobic exercise + weight loss. (3) Resistance exercise + weight loss. (4) Combination aerobic + resistance exercise + weight loss	IMAT and VAT via MRI; ISI via OGTT; physical function (modified physical performance test, VO2peak, gait speed); knee strength by dynamometry	Weight loss combined with both aerobic and resistance exercise (COMB) was most effective in reducing ectopic fat (IMAT and VAT) and improving physical and metabolic function in obese older adults. Combination exercise improved insulin sensitivity and physical performance more than aerobic or resistance exercise alone
Koschker et al., 2023 [19]	Germany	RCT	60	(1) RYGB surgery. (2) PELI	Change in peak VO₂ (ml/min/kg) via CPET; PFS of SF-36; body weight and composition; 6-minute walk test; left ventricular mass; mood (PHQ-9); comorbidities (hypertension, diabetes, etc.)	RYGB led to significantly greater improvements in cardiopulmonary capacity, weight loss, physical functioning, and quality of life compared to PELI. These changes were clinically relevant and sustained at 24 months
Vargas et al., 2023 [20]	USA	RCT	36	(1) ESG (n=18) performed with an overstitch device. (2) Moderate-intensity LS (n=18): reduced-calorie diet, 150 min/week aerobic exercise	Gastric emptying (T1/2) at 3 and 12 months; Weight loss (%TBWL) at 3, 6, 12 months; gastrointestinal hormones (ghrelin, GLP-1, PYY) at baseline and 12 months; eating behaviors (TFEQ-18) at baseline and 6 months; Gastric motility (subset, MRI) at baseline, 3, 12 months	ESG significantly delayed gastric emptying compared to LS at 3 and 12 months; greater delay in GE at 3 months correlated with greater weight loss. Gastric motility was preserved. Fasting ghrelin, GLP-1, and PYY increased after ESG. ESG promotes weight loss via delayed gastric emptying and hormonal changes while preserving motility, supporting its clinical adoption for obesity management
Wharton et al., 2023 [21]	Multinational (Canada, Italy, Hungary, Spain, and the United States)	RCT	174	Semaglutide 2.4 mg once weekly subcutaneous injection vs placebo, both plus LS modification (500 kcal deficit/day and 150 min physical activity/week) for 104 weeks	CoEQ scores at weeks 0, 20, 52, 104; body weight changes; food cravings; appetite; mood	Semaglutide 2.4 mg significantly improved control of eating, reduced food cravings, and led to substantial weight loss (~14.8% vs 2.4% placebo) over 2 years in adults with overweight/obesity. It improved both short- and long-term control of eating behaviors associated with weight loss
Cui et al., 2025 [22]	China	RCT	54	(1) Control - regular LS. (2) TRE - 10-hour eating window/day for 8 weeks. (3) RT - supervised resistance exercise 3x/week for 8 weeks. (4) TRE + RT combined	Body composition (weight, BMI, fat mass, fat-free mass, waist/hip circumference), blood pressure, mood status (anxiety, depression, stress), sleep quality	TRE alone reduced body weight and BMI but decreased fat-free mass; RT alone reduced fat mass and improved sleep quality; TRE + RT was most effective for weight/fat loss, maintaining muscle mass, and improving sleep quality without negative mood effects

Pharmacotherapy studies, including those by Pi-Sunyer et al. (2015) [11], Garvey et al. (2022) [17], and Wharton et al. (2023) [21], consistently show that glucagon-like peptide-1 (GLP-1) receptor agonists such as liraglutide and semaglutide significantly reduce body weight, improve glycemic control, and enhance quality of life over one to two years, though gastrointestinal side effects were common. Similarly, surgical approaches, such as in Kristensson et al. (2020) [14], Koschker et al. (2023) [19], and Vargas et al. (2023) [20], demonstrated substantial and sustained weight loss with added benefits for metabolic health and physical function, supporting their efficacy even when compared to enhanced lifestyle or psychological interventions.

Exercise-based interventions varied in intensity and structure. Chin et al. (2020) [13] and Berge et al. (2021) [15] showed that both high-intensity interval training (HIIT) and moderate-intensity continuous training (MICT) improved aerobic capacity and reduced fat mass, with low-frequency HIIT emerging as a feasible option. Waters et al. (2022) [18] further emphasized that combining aerobic and resistance training with weight loss strategies maximally improved ectopic fat reduction and insulin sensitivity, especially in older adults.

Dietary and behavioral strategies, such as TRE explored by Kotarsky et al. (2021) [16] and Cui et al. (2025) [22], demonstrated efficacy in reducing fat mass and improving lean mass, particularly when combined with resistance training. Probiotic use by Stenman et al. (2016) [12] and lifestyle-based approaches by Wharton et al. (2023) [21] further highlighted the role of gut microbiota and behavioral control in weight regulation.

Overall, this table illustrates that multimodal strategies, particularly those combining diet, exercise, pharmacology, and, in some cases, surgery, yield the most robust and sustained improvements in weight and metabolic outcomes. The effectiveness of these interventions also varies by population, adherence, and duration, underscoring the importance of individualized and comprehensive treatment plans for obesity and related conditions.

Critical Appraisal of Studies

**Figure 2:**
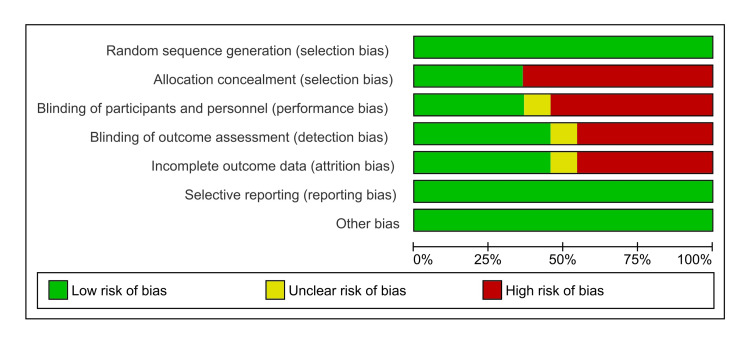
Risk of bias graph of the included studies evaluated using RoB 2

**Figure 3:**
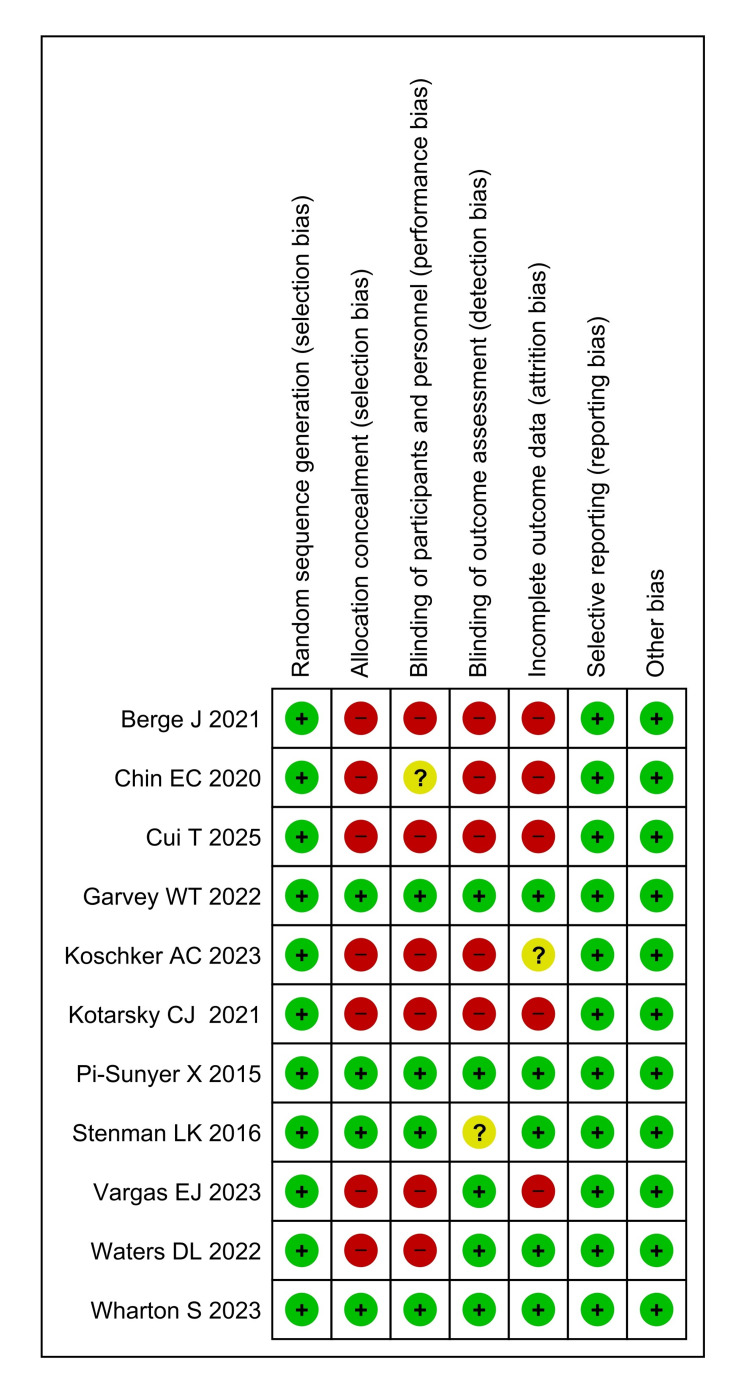
Risk of bias summary of the included studies assessed using RoB 2 [11-22]

Figure 2 and Figure 3 present a risk of bias assessment of 11 included RCTs using the Cochrane risk of bias assessment tool (RoB 2, The Cochrane Collaboration, London, UK), evaluating seven key domains: random sequence generation, allocation concealment, blinding of participants and personnel, blinding of outcome assessment, incomplete outcome data, reporting bias, and other bias. Among the studies, Pi-Sunyer et al. (2015) [11], Garvey et al. (2022) [17], and Wharton et al. (2023) [21] demonstrate the lowest overall risk of bias, scoring "low" in all categories, indicating high methodological rigor. Stenman et al. (2016) [12] also showed low risk in most areas, though the blinding of outcome assessment was marked as "unclear." Other studies like Chin et al. (2020) [13], Berge et al. (2021) [15], Kotarsky et al. (2021) [16], Koschker et al. (2023) [19], Vargas et al. (2023) [20], and Cui et al. (2025) [22] had higher risks of bias in multiple domains, particularly in allocation concealment, blinding, and handling of incomplete outcome data. For example, Chin et al. (2020) rated “high” in three domains (allocation concealment, blinding of outcome assessment, and incomplete outcome data), which may compromise the reliability of the findings. Similarly, studies from 2021 onward, including Berge et al. (2021) [15], Kotarsky et al. (2021) [16], and Cui et al. (2025) [22], tend to have more frequent high-risk judgments in these same areas. Studies of Waters et al. (2022) [18] and Koschker et al. (2023) [19] had mixed assessments, with "low" risk in some categories and "high" or "unclear" in others; these inconsistencies may reflect methodological limitations.

The Risk of Bias in Non-randomized Studies of Interventions (ROBINS-I) tool was used to appraise the quality of one study conducted by Kristensson et al. (2020) [14], which was non-randomized and was found to be of low risk of bias.

Discussion

This systematic review aims to explore recent trends in the prevention and management of obesity among adults. The findings of this study that pharmacological interventions such as liraglutide and semaglutide lead to significant and sustained weight loss and improved cardiometabolic risk factors in adults align with other published studies. The present study discussed only those pharmacological agents and other interventions used in the management of obesity that were reported by the analyzed studies based on the inclusion and exclusion criteria. A 2025 systematic review and meta-analysis of 11 randomized clinical trials involving 1,328 non-diabetic overweight or obese adults found that liraglutide significantly reduced body weight by an average of 4.59 kg compared to controls. It also improved waist circumference and BMI, though the effect on HbA1c was not significant in this non-diabetic population. This supports our finding of liraglutide’s efficacy in weight control and cardiometabolic risk factor improvement [23]. Another study on liraglutide 3.0 mg over 78 weeks demonstrated that while some weight regain occurred, fat mass loss was associated with sustained clinically significant weight reduction, indicating liraglutide’s long-term benefit in obesity management [24]. A 2022 meta-analysis by Gao et al. (2022) of eight RCTs with 4,567 obese or overweight patients without diabetes showed semaglutide induced a robust weight loss of approximately 10.09% of body weight, with significant reductions in BMI and waist circumference. Semaglutide demonstrated beneficial effects on various cardiometabolic markers, including improvements in blood pressure, C-reactive protein levels, and lipid profiles, thereby substantiating its positive impact on cardiovascular and metabolic health. Adverse effects were mainly gastrointestinal but acceptable [25]. A systematic review including 12 RCTs and six non-RCTs with over 10,000 obese/overweight adults without diabetes found that both liraglutide and semaglutide led to clinically relevant (≥5%) weight loss in 48.2-88.7% of participants, were well tolerated, and improved metabolic factors. This review noted semaglutide may be more effective than liraglutide in some studies, though many were in diabetic populations [26].

A study conducted by El Nekidy et al. (2025) [27] found no statistically significant difference in weight loss efficacy between liraglutide and semaglutide. However, semaglutide showed superior HbA1c reduction in mixed populations, including diabetic patients. Some studies suggest semaglutide may have greater weight loss efficacy than liraglutide, especially in diabetic populations, but our study and others in mixed or non-diabetic populations show comparable efficacy [26,27]. Weight regain after liraglutide treatment, although less than with lifestyle interventions alone, has been documented, indicating the chronic nature of obesity and the need for sustained management strategies [24]. Adverse effects, mainly gastrointestinal, are more frequent with these GLP-1 receptor agonists but are generally manageable and acceptable given the benefits [25,26].

Our study findings on exercise interventions and TRE corroborate with several recent studies, though some nuances and contrasts exist. Multiple meta-analyses and randomized trials confirm that both HIIT and MICT improve aerobic capacity (VO2max), body composition (weight, BMI, fat percentage), and cardiovascular risk factors such as blood pressure and cholesterol in adults with obesity [28,29]. HIIT frequently delivers comparable or superior benefits to MICT in a shorter timeframe, positioning it as an effective and time-efficient exercise option. For example, HIIT sessions can be shorter by about 9-10 minutes yet yield similar or superior improvements in VO2max and fat loss. After 12 weeks of training, weight loss was noticed in both types of exercises; HIIT decreased body weight by −5.7 kg ((−8.3 kg to −3.1 kg); p = 0.001) while MICT decreased it by −6.0 kg ((−9.0 kg to −3.0 kg); p<0001) without significant differences between the experimental groups. There was a reduction in BMI of −1.9 kg/m2 ((−2.7 kg/m2 to −1.0 kg/m2); p=0.001) in HIIT and −2.1 kg/m2 ((−3.2 kg/m2 to −1.1 kg/m2); p<0.001) in MICT with no difference between groups [28].

Some studies show no significant difference in weight loss or body composition changes between HIIT and MICT when energy expenditure is matched, suggesting that total caloric burn rather than intensity per se drives fat loss [29,30]. Health-related quality of life improvements appear similar between HIIT and MICT, indicating that both exercise types enhance well-being without a clear superiority of one over the other [31]. Combination therapies, such as weight loss programs incorporating both aerobic and resistance exercise, were found to be the most effective in reducing ectopic fat and improving physical and metabolic function [18].

Our study's findings, which show that bariatric surgery leads to significant weight loss, increased diabetes remission, and a reduced incidence of diabetes-related complications, align with previously published studies. Multiple meta-analyses and RCTs have shown that bariatric surgery leads to substantial and sustained weight loss compared to nonsurgical interventions, with procedures like GB and sleeve gastrectomy (SG) being particularly effective. For example, a comprehensive meta-analysis found that GB was more effective than adjustable gastric banding (AGB) and nonsurgical methods in reducing BMI and inducing weight loss [32]. Similarly, clinical trials report diabetes remission rates ranging from 33% to 90% at one-year post-surgery, significantly higher than medical management alone [33]. The Swedish Obesity Study (SOS) reported 72.4% diabetes remission at 2 years post-surgery versus 16.4% in controls [33]. Bariatric surgery not only improves glycemic control but also reduces the incidence of diabetes-related microvascular and macrovascular complications. The SOS 15-year follow-up showed a marked reduction in microvascular complications (20.6 vs. 41.8 per 1000 person-years) and macrovascular complications (31.7 vs. 44.2 per 1000 person-years) in surgical patients compared to controls, with hazard ratios of 0.44 and 0.68, respectively [33].

Additionally, long-term mortality rates are lower in patients undergoing bariatric surgery [33]. Bariatric surgery patients experience sustained improvements in glycemic control, often requiring fewer diabetes medications, including insulin. One study reported an 87% reduction in oral diabetes medication use and a 79% reduction in insulin use after surgery [33]. A recent pooled analysis of RCTs with up to 12 years of follow-up confirmed superior long-term glycemic control, increased diabetes remission, and improved lipid profiles in surgically treated patients compared to medical/lifestyle management, although with some increased risks of nutritional deficiencies and bone fractures [34]. Besides the efficacy of bariatric surgery in obesity management, a study by Vargas et al. (2023) [20] examined endoscopic sleeve gastroplasty, showing it significantly delayed gastric emptying and promoted weight loss via hormonal changes.

Despite potential advantages, bariatric surgery is not without its drawbacks, encompassing various potential complications, the necessity for subsequent reoperations, and the possibility of developing nutritional deficiencies. Complication rates range from 10% to 17%, and reoperation rates are about 7%, with mortality generally low but present (0.08-0.35%) [32]. Some studies also note increased risks of bone fractures and osteoporosis post-surgery [34,35]. The effectiveness and safety profiles vary by surgical type. GB tends to yield greater weight loss but higher complication rates compared to AGB, which has lower mortality but higher reoperation rates [32]. SG appears to have intermediate outcomes between GB and AGB but requires more evidence for long-term conclusions [32]. Some cohort studies may overestimate surgery benefits due to selection bias (patients opting for surgery may differ from those managed medically) and underestimation of medical therapy effectiveness if control groups lack rigorous weight loss programs [33].

Future Directions

Future research should focus on developing personalized obesity management strategies that integrate pharmacological, surgical, dietary, and exercise interventions tailored to individual patient profiles, including genetic, metabolic, and behavioral factors. There is a need for large-scale, long-term RCTs to evaluate the sustained efficacy and safety of newer pharmacological agents like semaglutide and liraglutide, as well as emerging dietary patterns such as TRE, especially in diverse populations. Research should aim to optimize combination interventions, particularly integrating various exercise modalities with dietary and pharmacological treatments, to maximize fat loss, preserve lean mass, and improve cardiometabolic outcomes.

## Conclusions

This systematic review highlights the multifaceted nature of obesity prevention and management among adults, emphasizing the effectiveness of diverse interventions. Pharmacological agents like liraglutide and semaglutide provide significant and sustained weight loss alongside cardiometabolic benefits. Exercise modalities, including HIIT and MICT, improve physical fitness and cardiovascular health, while TRE combined with resistance training supports fat loss and muscle preservation. Bariatric surgery remains a powerful intervention for substantial weight reduction and diabetes remission. Emerging evidence suggests probiotics may play a supportive role in weight management. Notably, combination therapies integrating aerobic and resistance exercise with dietary and pharmacological approaches yield the most comprehensive improvements in body composition and metabolic function. Continued research and tailored implementation of these strategies are essential to effectively combat the global obesity epidemic and its associated health burdens.
